# Research progress on the functional study of host resistance-related genes against *Heterodera glycines*

**DOI:** 10.1007/s44297-023-00008-7

**Published:** 2023-09-22

**Authors:** Long He, Nabi Noor Ul Ghani, Luying Chen, Qiannan Liu, Jingwu Zheng, Shaojie Han

**Affiliations:** 1 Laboratory of Plant Nematology, Institute of Biotechnology, College of Agriculture & Biotechnology, Zhejiang University, Hangzhou, 310058 Zhejiang China; 2 Ministry of Agriculture Key Laboratory of Molecular Biology of Crop Pathogens and Insects, Institute of Biotechnology, Zhejiang University, Hangzhou, 310058 China; 3 Key Laboratory of Biology of Crop Pathogens and Insects of Zhejiang Province, Zhejiang University, Hangzhou, 310058 China

**Keywords:** Soybean cyst nematode resistance mechanism, *rhg1*, *Rhg4*, Plant immune signaling molecules, Nematode-host interaction

## Abstract

Soybean (*Glycine max* L.), a crucial crop that provides essential nutrition, is experiencing increasing demand to meet protein and oil requirements. However, the menace of soybean cyst nematode (SCN) disease, caused by *Heterodera glycines*, poses a substantial threat globally, resulting in significant annual economic losses. While cultivating resistant varieties is an eco-friendly approach to control SCN, the excessive use of a single variety triggers ongoing evolution of SCN races, jeopardizing the soybean industry's stability. Leveraging advanced technologies, research on soybean SCN resistance mechanisms has progressed significantly across genetics, transcriptomics, and protein functions. This review consolidates insights into major resistance loci (*rhg1* and *Rhg4*), elucidating their connections with vesicle transport and plant hormone signaling pathways. It also discusses the role of key functional proteins in soybean resistance and addresses potential research issues. This study explores superior soybean resistance genes, laying a foundation for creating new SCN-resistant germplasms, thereby ensuring the sustainable growth of the global soybean industry.

**Supplementary Information:**

The online version contains supplementary material available at 10.1007/s44297-023-00008-7.

## Introduction

Cultivated for approximately 5000 years in China, soybean (*Glycine max* L.) stands as a vital leguminous crop, offering renewable vegetable protein and oil. Its cultivation has spread globally, encompassing diverse regions [1]. Beyond human consumption, soybeans are extensively used, particularly as animal feed [2]. In 2022, China emerged as the fourth-largest global soybean producer, yielding 20.28 million metric tons. However, an array of biotic and abiotic threats imperil soybean production, including pathogens such as viruses, bacteria, fungi, and nematodes [3]. Among these reported soybean pathogens, the soybean cyst nematode (SCN) stands out as a global menace, causing substantial yield losses. Its impact is profound, and effectively preventing and controlling SCN infection remains challenging [4].

SCN is a soil-borne, sedentary, and specialized parasitic nematode that has caused economic losses exceeding $32 billion in the United States alone between 1996 and 2016, averaging over $1.5 billion annually. SCN-induced losses exhibit substantial variability, ranging from 30–40% and even reaching as high as 100% in severely infested fields [5]. While there are excellent comprehensive recent reviews that cover SCN resistance in general [4–7], our focus in this review is specifically on the molecular-level aspects of SCN resistance. Generally, SCN is believed to originate in China or Japan [8]. Its first identification was reported in China in 1954. Since then, ten races of SCN have been documented in China. However, a recent discovery identified a new race (X12) in China capable of infecting all tested soybean sources and exhibiting higher virulence than the previously considered most virulent race, race 4 [9, 10]. As SCN has spread to many countries, such as the USA, Brazil and China, it has emerged as a significant problem, primarily due to the widespread areas of cultivated soybeans. The challenge in eradicating SCN, coupled with significant economic losses in soybean, further accentuate its impact. Common symptoms of SCN infestation include stunted growth, yellowing of plants, reduced seed production, and leaf loss. Infected rootlets also exhibit a decrease in bacterial nodules. Initially, the disease appears in patchy formations, and it takes approximately two years to fully infect a field.

**Fig. 1:**
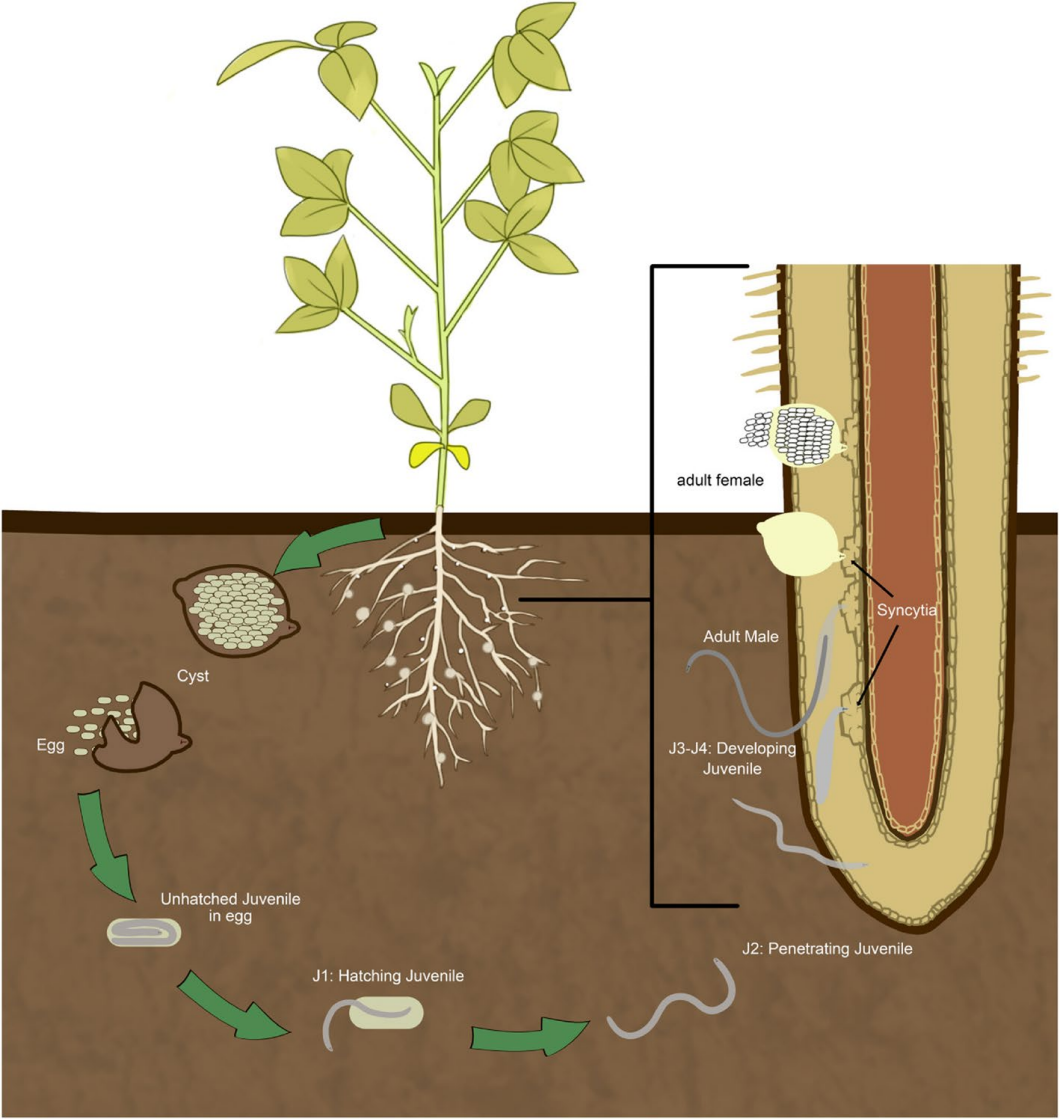
Life Cycle of Soybean Cyst Nematode (SCN). Schematic representation illustrating the life cycle of the soybean cyst nematode (SCN) in the root system of soybean plants. The diagram highlights the stages of nematode development and depicts the damaging effects on the plant, including nutrient depletion leading to yellowing of leaves. The cycle encompasses the various phases J0-J4 of nematode reproduction and infection, shedding light on the crucial interactions between SCN and soybean roots

The life cycle of the SCN can be divided into four stages, including juvenile stages and one adult stage (Fig. 1). The development begins with the eggs and progresses through molting. Second-stage juveniles penetrate the host roots and reach the vascular cylinder. By continuously feeding and secreting effectors, the second-stage juveniles (J2) of the nematode establish permanent feeding sites called syncytia. The nematodes transition to a sedentary lifestyle [6]. These feeding sites are formed in the vascular cylinder of the roots [11]. The interaction between the nematode and host can be either compatible or incompatible. In a compatible interaction, the nematode successfully completes its entire life cycle within the host, which typically takes approximately 24–30 days under optimal conditions.

Crop rotation and the implementation of resistant varieties are recognized as the most eco-friendly and economic measures among the diverse approaches to manage SCN. The utilization of resistant varieties, in particular, plays a pivotal role in achieving environmentally sustainable control strategies [6]. The application of resistant varieties, including *rhg1-b* derived from the resistant soybean variety PI 88788 and *rhg1-a* and *Rhg4* derived from PI 548402/Peking, along with other minor-effect quantitative trait loci (QTLs), such as *cqSCN-006* and *cqSCN-007* from PI 468916 and *Chr10-QTL* from PI 567516C, plays a crucial role in green control strategies. Over 300 SCN resistance-related QTLs have been identified through genome-wide association studies (GWAS) and population segregation analysis. These QTLs span all 20 chromosomes in soybean (Table S1) (https://www.soybase.org).

As an obligate parasitic nematode, SCN exhibits distinct physiological differentiation, and based on its varying pathogenicity on different host resistance backgrounds, it can be further divided into different physiological races [12]. With the continuous field application of single resistance loci, both domestically and internationally, SCN populations of different physiological races have evolved under the selection pressure of resistance, resulting in a reduction in the effectiveness of existing natural resistance genes [13–16]. With the limited and declining natural resistance in the face of potential resistance failure, it is crucial to conduct comprehensive research on the resistance mechanism of present resistance loci and explore high-quality targets for resistance genes in transgenic soybean varieties. This has become a challenging yet vital focus of environmentally sustainable control of SCN globally. In recent years, significant breakthroughs have been made in the exploration of SCN resistance-related genes in soybean, shedding light on their physiological and biochemical functions and enhancing our understanding of resistance mechanisms. This article provides an overview of the recent advancements in functional research on soybean resistance-related genes against SCN, while the prospects and future directions for further exploration in this field are also discussed.

## Research on the gene functions of soybean's QTL loci against SCN

Due to the unique characteristics of plant-parasitic nematodes, their interaction mechanisms differ significantly from currently known mechanisms of other pathogens, including fungi, bacteria, and viruses. One impressive example is the absence of traditional pathogen-associated molecular pattern (PAMP) receptors or intracellular nucleotide-binding-leucine-rich-repeat (NLR) receptors encoded by known SCN resistance loci. Further studies indicate that LRR-RKL kinases located near significant quantitative trait loci (QTLs) do not confer resistance to SCN [17, 18]. These findings demonstrate that SCN may exhibit more vigor and proactivity than other pathogens due to its unique properties, and the recognition mechanism of SCN differs from that of other conventional plant‒microbe immune activation pathways.

In recent years, with the application of technologies such as soybean gene chips, next-generation transcriptome sequencing, and laser microdissection, an increasing number of potential SCN resistance-related genes have been identified, along with their functions [19–27]. In particular, by the use of a nematode-adapted single-cell RNA-seq approach, novel virulence gene candidates have been uncovered within SCNs [28, 29]. Stable T2 generation mutant lines are obtained through ethylmethanesulfonate (EMS) mutagenesis of susceptible or resistant varieties, followed by traditional targeting-induced local lesions in genomes (TILLING) or TILLING combined with second-generation sequencing technology, which is an effective method for screening and discovering potential resistance-related genes [30–32]. Currently, as the study of known SCN resistance genes deepens, the mechanism of interaction between SCN and soybean is gradually being elucidated.

Among the QTLs linked to SCN resistance, *rhg1* and *Rhg4* are of paramount importance. In the following sections, we provide a detailed introduction to the functions of the genes encoded by these two major-effect resistance loci, their gene structure characteristics, and their collaborative mechanism.

### *rhg1*

*Rhg1* (*Resistance to Heterodera glycines 1*) is the earliest identified major resistance locus derived from the classical cultivar PI 88788, located on chromosome 18. According to statistics, more than 95% of the SCN-resistant cultivars in the United States carry the *rhg1* locus from the PI 88788 line, indicating the stability and practicality of this locus in providing resistance [33].

Cook et al. (2012) identified *rhg1* as a 31.2 kb gene segment using fluorescence in situ hybridization (FISH), and the multicopy nature of *rhg1* is directly associated with SCN resistance. The study unveiled that susceptible cultivars (e.g., Williams 82) possess a single *rhg1* copy, termed the *rhg1*-*c* type. Resistant cultivars (e.g., PI548402/Peking) display 2–3 tandem copies, identified as the *rhg1*-*a* type. Notably, the representative resistant cultivar PI 88788 features 7–10 tandem copies recognized as the *rhg1*-*b* type [34]. Recently, a new *rhg1* haplotype, called *rhg1-ds*, was discovered in six accessions of wild-type soybean, *Glycine soja* Siebold & Zucc, using SoySNP50K data and assessing the presence of the NSF_RAN07_ allele [35]. With the rapid development of pangenome sequencing technology, multiple copies of genes in soybean have been found to be widespread and are thought to be indispensable for soybean evolution in nature [36]. Transcriptional analysis of the *rhg1*-encoded resistance genes in soybean samples with different copy numbers showed that under noninfected conditions, transcript abundance for these genes scales with *rhg1* copy number. In addition, quite a few genetic structural variations, including single-nucleotide polymorphisms (SNPs), DNA fragment insertions, and deletions, are observed in these genes encoded by different types of *rhg1* in soybean (Cook et al., 2014). The effects of genetic structural variations of genes encoded by *rhg1* on SCN resistance are still under investigation.

Three out of the four genes encoded by the *rhg1* locus have been demonstrated to engage in the resistance response of *rhg1* high-copy cultivars against SCN [34]. These three genes are as follows: *Glyma.18G022400* (Wm82.a1, Glyma18g02580), which encodes a putative amino acid transporter protein called GmAAT; *Glyma.18G022500* (Wm82.a1, Glyma18g02590), which encodes a functionally conserved protein α-SNAP (α-soluble NSF attachment protein, also known as SNAP18) involved in vesicular transport processes; and *Glyma.18G022700* (Wm82.a1, Glyma18g02610), which encodes a protein WI12_Rhg1_ (WI12-induced protein) containing a WI12 injury-induced structural domain. When these *rhg1* genes from soybean sources were transferred into *Arabidopsis* or *Solanaceae* (potato), enhanced resistance against sugar beet cyst nematode (*Heterodera schachtii*), potato cyst nematode (*Globodera rostochiensis*), and potato pale cyst nematode (*G. pallida*) was observed [37].

The functional mechanism of α-SNAP encoded by the *rhg1* locus against SCN is well characterized [38–40]. α-SNAP is a conserved protein involved in vesicular transport processes and forms a complex with N-ethylmaleimide-sensitive factor (NSF) to participate in the recycling of soluble NSF attachment protein receptors (SNAREs). α-SNAP specifically accumulates in the feeding sites called syncytia of SCN during nematode infection [38, 41]. Different soybean varieties with varying copy numbers of *rhg1* encode α-SNAP with amino acid polymorphisms, particularly in the C-terminal region, which affects its interaction with NSF and indirectly influences intracellular vesicular transport mechanisms. Overexpression of *α-SNAP* encoded by high-copy *rhg1* can lead to plant cell death, while α-SNAP from susceptible varieties does not cause such effects [38]. However, *α-SNAP* itself is an essential gene for cellular processes, and mutations occurring in resistant types of *α-SNAP* pose a destructive threat to plant viability. To balance the toxicity of the *α-SNAP* mutant in resistant varieties, almost all known soybean varieties with resistant α-SNAP have coevolved with a stronger binding partner called NSF_RAN07_, which ensures that normal cellular functions are not compromised [39]. Different mutations in *α-SNAP* result in distinct physiological functions, indicating that *rhg1* affects resistance not only through copy number variation but also through intrinsic genetic sequence variation [40, 42]. The homologous gene of *α-SNAP*, *SNAP11* (Glyma.11G234500), on chromosome 11 has been identified as a minor-effect gene involved in SCN resistance and contributes to the resistance of Peking-type soybean hosts against SCN [43, 44]. While not yet conclusively proven, the significance of α-SNAP in cyst nematode resistance suggests its vulnerability to nematode effectors that could potentially dampen host defense against cyst nematodes through α-SNAP-mediated mechanisms. An SCN gene encodes a bacterial-like protein, HgSLP-1, containing a putative SNARE domain, which physically interacts with α-SNAP in vitro [45]. Recently, an effector named HsSNARE1, containing a t-SNARE domain, was identified from beet cyst nematode (BCN). HsSNARE1 and its highly homologous counterpart HgSNARE1 from the SCN can both interact with AtSNAP2, which is highly homologous to GmSNAP18. Overexpressing *HgSNARE1* suppresses BCN infection, while *HsSNARE1* overexpression promotes it in *Arabidopsis thaliana* [46]. Further exploration is required to identify additional nematode effectors that directly target GmSNAP18/α-SNAP.

The mechanism underlying another resistance gene, *GmAAT*, encoded by the *rhg1* locus, remains inadequately comprehended to date. There are no amino acid sequence differences among different *rhg1* haplotypes for GmAAT, suggesting that the resistance mechanism of *GmAAT* is quite distinct from that of the *α-SNAP* gene. Silencing *GmAAT* leads to compromised soybean resistance against SCN [34]. GmAAT contains a conserved amino acid transport domain, but there is no direct evidence showing that this protein directly transports amino acids in soybean. Overexpression of *GmAAT* increases soybean tolerance to excess glutamate, indicating its involvement in glutamate tolerance-related functions [47]. Furthermore, overexpression of *GmAAT* can activate the jasmonic acid signaling pathway, indicating that *GmAAT* potentially operates by stimulating plant hormone pathways [47].

Using immunoelectron microscopy with a GmAAT-specific antibody, specific expression and localization patterns of GmAAT in response to SCN infection were observed. During the early stages of SCN invasion in the root system, GmAAT protein specifically accumulates in cells penetrated by SCN and forms resistance vesicles with GmAAT localization in cells directly contacted by the nematode. The copy number of *rhg1* is positively correlated with the enrichment of GmAAT in these structures, and the expression level of *GmAAT* and the extent of vesicle aggregation are both associated with *rhg1*-mediated resistance [48]. The physiological and biochemical properties of resistance vesicles formed by GmAAT and the molecular mechanisms of GmAAT's involvement in other plant hormone signaling pathways, such as JA and ethylene, still require further investigation. Collectively, current research suggests that while *GmAAT* and *α-SNAP* both belong to the *rhg1* locus, their resistance mechanisms significantly diverge in terms of protein localization and physiological function.

*WI12*_*Rhg1*_ is the third resistance gene encoded by the *rhg1* locus, and it has not garnered significant research attention thus far. In addition, there is a lack of studies on homologous proteins of WI12_Rhg1_ in other species. A recent study showed that WI12_Rhg1_ directly interacted with DELLA18 (Glyma.18G040000) in yeast and plants. Double knockout of *DELLA18* and its homeolog *DELLA11* (Glyma.11G216500) leads to a significant reduction in SCN resistance and causes changes in root morphology [49]. Due to its brief protein sequence, uncovering valuable functional domains within WI12_Rhg1_ proves to be challenging. The specific physiological function of WI12_Rhg1_, which contains a putative damage-inducible domain, also needs to be confirmed. However, one potential resistance mechanism of WI12_Rhg1_ likely involves damage-associated disease progression molecular patterns (wound-inducible DAMPs) through interactions with other proteins, including those encoded by the *rhg1* locus. This mechanism may contribute to the early response of SCN infection during plant-nematode interactions.

### *Rhg4*

*Rhg4* is located on chromosome 8 and is one of the major QTLs derived from Peking and PI 437654 [50–52]. Liu et al. identified the *serine hydroxymethyltransferase* (*SHMT* or *SHMT08*) gene located at the *Rhg4* locus using TILLING technology, a commonly employed reverse genetics method. *shmt* mutations can impact the resistance of Peking-type resistant cultivars [32]. Similar to α-SNAP, SHMT is a housekeeping protein and is involved in the folate synthesis signaling pathway. The sequence of *SHMT* genes exhibits only five variations between wild-type and Peking-type resistant varieties; however, these variations profoundly impact SCN resistance. The underlying mechanisms linking the mutation sites to the phenotypes still require further research. It is worth noting that, similar to α-SNAP, SHMT is specifically expressed in the syncytial cells formed during SCN infection [32]. Genetic screening has provided further validation of the structure and resistance function of *SHMT* [53]. Through protein‒protein interaction screening, the identification of a 70-kDa heat shock protein (HgHSP70) in *H. glycines* revealed its interaction with GmSHMT08. These studies have provided insights into the interaction mechanism between soybean and SCN [54].

Similar to *rhg1*, a recent whole-genome sequencing study discovered that *Rhg4* also undergoes multicopy amplification [55]. A tandem repeat sequence of approximately 35.7 kb exists within *Rhg4*, and high-level amplification of *Rhg4* can lead to increased transcription levels of the genes encoded by *Rhg4*. This segment of the *Rhg4* locus contains three genes: *Glyma.08g108800* (Wm82.a1, Glyma08g11480), *Glyma.08g108900* (SHMT, Wm82.a1, Glyma08g11490), and *Glyma.08g109000* (Wm82.a1, Glyma08g11500). Additionally, *Rhg4* also exhibits allelic variation and is primarily categorized into *Rhg4-b* (single copy) represented by the wild type and *Rhg4-a* (1–4.3 copies) represented by the resistant Peking-type [55]. The promoter sequence of *SHMT08* in resistant varieties such as Peking and PI 88788 differs from that in susceptible varieties and is closely associated with a broader spectrum of SCN resistance [55].

In resistant varieties such as PI548655 (Forrest, a Peking-type resistant variety) and PI 88788, the transcription levels of *SHMT08* are approximately twofold higher during early SCN infection compared to that in wild type [56]. This indicates that the resistance-type promoter may play a role in early infection regulation at the transcriptional level. The genomic studies of *rhg1* and *Rhg4*, combined with the functional genomics of SCN resistance phenotypes, once again demonstrate that soybeans generally adapt to the invasion of external pathogens by multicopy amplification and sequence structural variations at specific gene loci. The association between gene changes and SCN resistance, particularly the expression regulatory mechanism, still requires further research.

### Diverse mechanisms and associations of *rhg1* and *Rhg4*

Although *rhg1* and *Rhg4* are both important resistance loci, their resistance mechanisms are quite different. They share significant associations and similarities, mainly manifested in the following aspects. First, the functional proteins SHMT08 and α-SNAP encoded by these two loci interact with each other and coexist in a protein complex. [57]. Overexpressing soybean *GmSNAP18* in *Arabidopsis* increased its susceptibility to BCN. Transcriptome analysis showed that *GmSNAP18* overexpression in *Arabidopsis* led to the suppression of *AtSHMT4*, a homolog of *GmSHMT08*, post BCN infection. This reveals the adverse negative modulation of *AtSHMT4* in BCN susceptibility by *GmSNAP18* overexpression [58]. However, the detailed function and mechanism of their interaction are still unknown. Second, when the copy number of *rhg1* is less than 5.6, it cannot provide sufficient broad-spectrum SCN resistance independently and requires cooperation with *Rhg4*. The combination of low-copy *rhg1* and resistance-associated *Rhg4* is collectively referred to as Peking-type resistance (*rhg1-a* + *Rhg4*-a/*Rhg4*-c) [55, 59]. The specific mechanism of this cooperation needs further investigation. Third, as evolution progresses, both *rhg1* and *Rhg4* in resistant varieties have multiple homologous genes in different resistance types. This is evident in the abundance of nonsynonymous single nucleotide polymorphisms (SNPs) causing amino acid sequence variations. The structural and functional changes resulting from these variations require further study. Fourth, both loci exert additional resistance functions through multicopy amplification. Higher copy numbers of *Rhg4* provide broader resistance against various virulent races of SCN, while increased copy numbers of *rhg1* independently provide sufficient resistance. Fifth, based on whole-genome resequencing of 106 soybean lines, specific mutations in the promoter and gene sequences of *rhg1* and *Rhg4*, as well as differences in copy numbers, collectively determine resistance to different physiological races of SCN [55]. The coevolutionary relationship between these resistance genes and the pathogen deserves further investigation.

## Vesicle transport and autophagy in SCN resistance

### Vesicle transport in SCN resistance

Vesicle transport is a specific and dynamic process involving the translocation of cargo vesicles from the donor membrane to the target membrane. This process includes complex events such as endocytosis and exocytosis, which require a range of conserved proteins [60]. Vesicle transport plays a significant role in plant immunity. Resistance proteins in the extracellular space are secreted through this process to exert their functions. Additionally, the endocytosis of resistance molecule receptors on the plant's surface can significantly activate downstream signaling pathways. Proteins involved in vesicle transport pathways are often targeted by pathogens to interfere with plant resistance [61].

The exocyst, acting as a receptor protein, plays a pivotal role in signal transduction. The exocyst serves as the "bridge" between incoming vesicles and receptor proteins, leading to the aggregation of SNARE protein complexes and membrane fusion. The exocyst complex is an octameric structure, and a study in yeast identified eight protein components: Sec3p (EXOC1), Sec5p (EXOC2), Sec6p (EXOC3), Sec8p (EXOC4), Sec10p (EXOC5), Sec15p (EXOC6), Exo70p (EXOC7), and Exo84p (EXOC8). Each component of the exocyst complex has its own molecular mechanism involved in plant resistance. The exocyst protein PR-1 (Glyma.15g062400) has been demonstrated to directly participate in SCN resistance in soybean, indicating the involvement of the exocyst pathway in SCN resistance [62]. Sec4p physically interacts with Sec15p (EXOC6), which can regulate the assembly of exocyst vesicles. Overexpression of *Sec4* can also enhance SCN resistance in soybean [63]. All homologous genes of each component of the exocyst complex have been identified in soybean. Additionally, the expression levels of each component gene were significantly upregulated in the syncytia, indicating that exocyst vesicles contribute to SCN resistance in soybean. Overexpression of exocyst component genes in the susceptible cultivar Williams 82 enhances resistance, while silencing of the related genes in the resistant cultivar Peking renders the plants more susceptible [64].

SNARE proteins mediate vesicle fusion, which is a crucial step in vesicle transport. After membrane fusion, the dissociation and recycling of SNAREs require NSF and α-SNAP proteins, which together with SNAREs form a supercomplex called the 20S complex. This complex is involved in the recycling process of vesicle transport components [65]. Transcriptional analysis of SCN-infected root cells reveals that the expression of genes encoding the 20S complex is induced [62]. As mentioned above, *α-SNAP i*s a key SCN resistance gene encoded by the *rhg1* locus. Once more, these findings underscore that SCN can potentially target vesicle fusion components to facilitate susceptibility.

Synaptic fusion protein (syntaxin, SYN) is a part of the SNARE protein complex, which means that syntaxin interacts physically with α-SNAP protein. In soybean, overexpression of *α-SNAP* induces the expression of *SYN31*, and the upregulation of SYN31 predominantly occurs within cytoplasmic cells upon SCN infection. Further study shows that overexpression of *SYN31* can confer SCN resistance in susceptible soybean roots [66]. Overexpression of *SYN121,* the homologous gene in soybean of *Arabidopsis PEN1*, can also enhance SCN resistance in soybean. The transcription levels of *SYN121* and other genes encoding the SNARE complex are upregulated in SCN-infected soybean cells [67]. Recent research has demonstrated that α-SNAP interacts with two syntaxin proteins (SYN12, Glyma.12g194800, and SYN16, Glyma.16g154200) belonging to the t-SNARE family. Both of these syntaxin proteins are reported to be located within SCN resistance QTLs. Knocking out two *syntaxin* genes using CRISPR technology in the Peking resistant soybean variety compromises resistance [68]. Another protein complex closely associated with SNARE in the 20S complex, COG, is involved in regulating membrane fusion and has been reported to directly influence the SCN resistance response. Overexpression of the *COG* gene significantly inhibits SCN parasitism, and silencing the *COG* gene in the Peking resistant variety significantly affects resistance [69]. In conclusion, vesicle transport plays a crucial role in SCN pathogenicity and host resistance, but more direct evidence and the detailed mechanism of vesicle transport still need to be explored.

### Autophagy in SCN resistance

Plant autophagy, a conserved intracellular degradation system, is gaining increasing recognition as a crucial player in plant‒pathogen interactions, yet its involvement in nematode parasitism remains largely unexplored [70]. It was demonstrated that disrupting autophagy in *Arabidopsis* mutants led to reduced susceptibility to *Heterodera schachtii* infection, highlighting the pivotal role of autophagy in plant-nematode interactions [71]. Recently**,** research conducted by Zou et al. underscores the significance of autophagy in bolstering jasmonate-driven defenses in plants against nematode challenges [72]. As elaborated above, vesicle transport is pivotal in SCN pathogenicity and host resistance. Additionally, vesicle transport contributes to autophagy by facilitating autophagosome formation. It is plausible to suggest that autophagy could contribute to defense against SCN, with the nematode potentially disrupting host vesicle trafficking to aid infection. However, direct evidence is still lacking.

## Signaling molecules in SCN resistance

The resistance mechanisms of plant hosts against various pathogens differ, and these mechanisms typically involve the transduction of signals related to plant disease resistance. Cellular signaling is the process by which cells receive initial stimulus signals that are subsequently transmitted and amplified. Plant hormones represent a significant category of signaling molecules that play a crucial role in plant immunity. These include auxins, ethylene, cytokinins, gibberellins, abscisic acid, jasmonates, salicylic acid, brassinosteroids, and strigolactones. Other nonhormonal signaling molecules produced by plant cells function as defense regulators, primarily encompassing small peptides, reactive oxygen species (ROS), calcium ions (Ca^2+^) and more. Transcriptomic analyses have demonstrated that numerous genes encoding plant immune-related signaling molecules are influenced by nematode infection. The involvement of plant signaling molecules in nematode resistance has been a prominent subject within this research domain.

### Plant hormone molecules in SCN resistance

#### Ethylene in SCN resistance

The ethylene signaling pathway is crucial for plant root development, but its involvement in SCN resistance is largely unknown. Hu et al. reported that the ethylene signaling pathway is involved in regulating root attraction to SCN, and soybean roots treated with ethylene inhibitors are more attracted to SCN [73]. Additionally, the expression levels of genes encoding the ethylene precursor ACC synthase were upregulated upon SCN infection [74]. Transcription factors downstream of the ethylene signaling pathway have been demonstrated to undergo upregulation following SCN infection. As an example, the expression of *GmEREBP* (Glyma.18G252300), an ethylene-responsive transcription factor that binds to the GCC-box motif, is downregulated in susceptible soybean varieties and upregulated in resistant soybean varieties [75]. Subsequent research suggests that the ethylene pathway may regulate ethylene-responsive genes, such as *PR3* and *PDF1.2*, through these transcription factors upon SCN infection [76]. Therefore, the interplay between the genes encoding resistance (such as *rhg1* and *Rhg4*) and the ethylene signaling pathway in susceptible or resistant soybean varieties remains to be explored.

#### Auxin in SCN resistance

Currently, investigations into the role of the auxin signaling pathway in SCN resistance remain limited. The expression of the auxin-responsive gene *ADR12* was found to be downregulated in soybean roots upon SCN infection [77]. Transcriptomic profiling of SCN-infected root cells in soybean confirmed that the expression of the genes related to auxin signaling pathways, such as IAA and ARF, was significantly regulated [20]. While it is acknowledged that sedentary plant-parasitic nematodes, such as root-knot nematodes and sugar beet cyst nematodes, utilize diverse strategies to alter auxin homeostasis [78–81], concrete evidence for the involvement of auxin signaling in SCN resistance has yet to be established.

#### Salicylic acid in SCN resistance

Salicylic acid (SA) plays a pivotal role and becomes activated during the initial phase of pathogen invasion. Soybean salicylic acid methyltransferase (GmSAMT1) has been identified as a contributor to SCN resistance [82]. Overexpression of *GmSAMT1* in susceptible varieties significantly inhibits nematode development and enhances SCN resistance. In soybean roots that overexpress *GmSAMT1*, the expression of SA signaling pathway-related genes (*GmNPR1*, *GmICS1*) is also significantly induced [82]. Therefore, it is predicted that the accumulation of the biosynthetic enzyme GmSAMT1 is faster in resistant varieties, which can activate the SA signaling pathway and contribute to SCN resistance.

### Nonhormonal signaling molecules in SCN resistance

The CLAVATA receptor complex, which plays a crucial role in shoot apical and root apical meristem development, is regulated by a small peptide called CLV3, which consists of a 12-amino acid peptide and belongs to the *CLE* (*Clavat Like Elements*) family. Plant-parasitic nematodes secrete CLE-like effectors to manipulate plant root development and facilitate their infection. In soybean, it has been demonstrated that the CLV3 receptor protein can specifically bind to CLE-like effectors secreted by SCN, and silencing this receptor leads to increased resistance against SCN [83]. Structural and functional analysis of SCN CLE-like effectors has identified a specific domain responsible for transporting these effectors from the endoplasmic reticulum to the extracellular space. Nematode CLE effectors can hijack the plant cell secretory system to export their signaling proteins outside the cell [84]. In addition, plant elicitor peptides (PEPs) have been shown to participate in regulating SCN resistance. Treatment of soybean seeds with PEP1, PEP2, and PEP3 significantly inhibits SCN proliferation. Further experiments have demonstrated that PEP treatment can induce the production of reactive oxygen species (ROS) in soybean, leading to SCN resistance [85]. Exploring the precise molecular mechanisms of plant elicitor peptide-induced resistance holds significant potential.

Extensive research has delved into the systemic response of plants to pathogen infection and wounding, revealing a network of compounds and signals involved [86]. Nematodes can induce physical damage during their early infection stages, yet the extent to which ROS and Ca^2+^ signaling mechanisms play a role in orchestrating this response remains largely unexplored. ROS are frequently generated in response to biotic or abiotic stress in plants, serving as essential components in various signaling pathways. RBOHC2, a key factor in plant ROS production, physically interacts with GmAAT encoded by *rhg1* in vesicles. Coexpression of RBOHC2 and GmAAT induces the production of superoxide anions (O^2−^), which serve as ROS precursors in plant cells. The O^2−^ generated around the nematode-containing vesicles can be further converted into hydrogen peroxide (H_2_O_2_) outside the plant cells, directly affecting the nematode or acting as a secondary signaling molecule to induce stronger downstream resistance responses [48]. Chen et al. also observed that the rate and extent of ROS accumulation varied greatly among different types of resistant varieties, and ROS production is directly related to host resistance against SCN [87]. Overexpression of *miR408* in soybean can decrease soybean resistance to SCN by suppressing ROS accumulation [88]. Ca^2+^ and ROS signaling interplay significantly in plant immunity. Ca^2+^ plays a pivotal role in signaling events during the invasion of the potato cyst nematode *Globodera rostochiensis* [89]*.* The MeTCTP effector from *Meloidogyne enterolobii* impedes immunity by suppressing cytoplasmic free Ca^2+^ through secretion [90]. No reports have indicated direct engagement of Ca^2+^ signaling pathways in SCN resistance; further attention is required to understand the relationship between ROS and Ca^2+^ signaling pathways and SCN resistance.

## Other mechanisms

Host cell wall degradation is prominent during syncytium formation. Many nematode secreted effectors are believed to have cell wall-degrading enzyme activity. However, the involvement of the host’s cell wall synthesis pathway in SCN resistance has been less studied. Recently, the biochemical enzyme GmXTH43, which is involved in the regulation of cell wall synthesis and the elongation of glycan side chains, was found to be specifically expressed in syncytial cells. Overexpression of *GmXTH43* in susceptible soybean varieties can enhance resistance, suggesting that it may function in syncytia by affecting cell wall formation [91]. This is another example of a fundamental biochemical enzyme involved in the response to SCN resistance, but the underlying regulatory mechanism remains unclear.

In addition, recent research has reported that overexpression of a plasma membrane-localized broad-spectrum resistance gene, *disease resistance 1* (*GmDR1*, Glyma.10g094800), can enhance plant resistance to SCN, aphids, and fungal diseases. However, the specific regulatory mechanism is still unknown [92].

In the context of amino acid metabolism and the SCN resistance mechanism mediated by *rhg1* and *Rhg4*, several intriguing questions arise. *Rhg4*, which encodes a *serine hydroxymethyltransferase* (*SHMT08*) involved in cellular one-carbon metabolism, plays a crucial role in interconverting serine and glycine. On the other hand, it has been suggested that *rhg1*, encoding the *GmAAT* transporter, may function as an amino acid transporter. However, the specific amino acid(s) transported by GmAAT and its potential connection to the resistance loci remain unclear. It is hypothesized that GmAAT transports glycine, which can serve as a substrate for *SHMT08* encoded by *Rhg4*. Moreover, the relationship between the amino acid pool and the plant hormone response signaling pathway upon SCN infection needs to be explored. A recent study found that the addition of specific free amino acids from an external source had a significant ability to attract second-stage juveniles (J2) of SCN in chemotaxis assays. Interestingly, it was observed that nine particular amino acids showed strong attraction to SCN J2 [93]. One possible explanation for this phenomenon is that the upregulation of *GmAAT* expression upon SCN infection may subsequently impact the balance of free amino acids within the apoplast of SCN-infected cells. This mechanism may serve as a defense strategy to deter the attraction of newly hatched J2s to soybean roots. Enzymes encoded by the *GH3* family can conjugate jasmonates, auxins, and benzoates to amino acids. This conjugation process has diverse effects, including the activation, inactivation, or degradation of these hormone molecules. By conjugating amino acids to plant hormones, plants have evolved a sophisticated immune system in the face of SCN infection and other environmental stimuli. Thus, through the examination of the interplay between amino acid homeostasis and the modulation of plant hormone responses, a new strategy for enhancing resistance in soybean varieties may potentially be uncovered. Further investigation into the relationship between these two resistance loci, *rhg1* together with *Rhg4*, and the amino acid pool and exploration of the impact of conjugated plant hormones on SCN infection holds great potential for gaining a deeper understanding of the complex mechanisms underlying SCN resistance.

In contrast to other plant microbiome pathogens, plant parasitic nematodes exhibit characteristics similar to animals and often rely on vitamins to support their parasitic activities. On the other hand, the ability of the plant host to regulate vitamin supply during the plant-nematode interaction may play a crucial role in facing cyst nematode infection. *SHMT08,* encoded by *Rhg4,* is a widely conserved enzyme regulating one-carbon folate metabolism across different kingdoms. It is speculated that folate (a natural form of vitamin B9) deficiency resulting from disrupted folate homeostasis can lead to significant resistance derived from Rhg4. A recent study conducted on sugar beet cyst nematode, *H. schachtii*, revealed an atypical completion of vitamin B5 biosynthesis by the parasitic animal [94]. The specific investigation of the resistance mechanisms associated with the balance of vitamin supply during SCN infection, as well as the study of plant-related genes involved in the modulation of disease resistance and susceptibility in this process, represents a promising avenue for future research in this field.

## Summary and outlook

Significant progress has been made in the functional study of soybean resistance genes against SCN, but there are still several limitations. Currently, transcriptomics and functional genomics analyses have identified a range of resistance genes, while research on expression and epigenetic regulation is lacking. Additionally, potential resistance genes are often essential for plant life processes, and silencing them can lead to defects in root development and growth, indirectly affecting resistance phenotypes. Discovering new nematode resistance genes involves utilizing modern techniques such as comparative genomics, expression quantitative trait locus (eQTL) mapping, association studies, gene editing, and omics technologies to identify candidate genes linked to nematode resistance. Functional validation and network analysis further validate their roles and interactions, enabling the development of resistant soybean varieties.

The detailed relationship between known plant immunity and SCN resistance genes has not been elucidated. Further research is needed to explore the differences and connections between PAMP-triggered immunity (PTI) and the wounding defense response induced by SCN. Additionally, it is important to investigate whether nematode effectors secreted into host cells can trigger effector-triggered immunity (ETI). Screening for interacting proteins using known important resistance-associated proteins as baits in the soybean system can help answer these questions and provide a better understanding of the interaction pathways between SCN and soybean hosts.

China, the birthplace of soybean, boasts extensive germplasm resources. Wild soybeans (*Glycine soja*) can serve as a valuable gene pool that likely contains abundant resistance genes different from those present in current cultivated varieties. Further exploration of resistance genes using wild soybeans is needed. Additionally, with the rapid advancement of single-cell sequencing technology, transcriptional profiling analysis can pinpoint key developmental nodes at the single-cell level. Moreover, it is essential to construct more intricate profiles through single-cell sequencing in both compatible or incompatible interactions between SCN and soybean. This approach can enhance our understanding of SCN pathogenicity and the host's resistance defense response.

Last, research on pathogenicity centered around nematodes is equally important, especially in the identification and functional verification of nematode effectors. The classification and identification of SCN-secreted biochemical signaling molecules such as ascarosides, as well as the development and application of gene manipulation techniques targeting plant-parasitic nematodes, hold great potential for development and application in plant breeding and scientific research.

The study of SCN resistance mechanisms contributes to understanding the interaction between plant-parasitic nematodes and hosts, providing references for traditional breeding and valuable targets for the construction of resistant transgenic cultivars. This study has significant implications for China's independent intellectual property rights in related breeding and scientific research fields. With the development of biotechnology and continuous investment in science and technology in China, significant breakthroughs are expected in the comprehensive and environmentally friendly control of soybean cyst nematodes in theory, research, and practical applications. Emerging theories, concepts, and practices will have a vital role in safeguarding the security of global soybean production and ensuring the safety and sustainability of the soybean industry worldwide.

## Supplementary Information

**Additional file 1: Table S1.** SCN-related QTLs in soybean.

## Data Availability

All data used in this study are included in this article.
